# VectorMOD: Method for Bottom-Up Proteomic Characterization of rAAV Capsid Post-Translational Modifications and Vector Impurities

**DOI:** 10.3389/fimmu.2021.657795

**Published:** 2021-04-01

**Authors:** Neil G. Rumachik, Stacy A. Malaker, Nicole K. Paulk

**Affiliations:** ^1^ Ion Chromatography and Sample Preparation, Thermo Fisher Scientific, Sunnyvale, CA, United States; ^2^ Department of Chemistry, Yale University, New Haven, CT, United States; ^3^ Department of Biochemistry & Biophysics, University of California San Francisco, San Francisco, CA, United States

**Keywords:** AAV (adeno-associated virus), PTM (post-translational modification), proteomics, mass spectrometry - LC-MS/MS, bottom-up approach, glycosylation, immunogenicity, adverse drug reaction

## Abstract

Progress in recombinant AAV gene therapy product and process development has advanced our understanding of the basic biology of this critical delivery vector. The discovery of rAAV capsid post-translational modifications (PTMs) has spurred interest in the field for detailed rAAV-specific methods for vector lot characterization by mass spectrometry given the unique challenges presented by this viral macromolecular complex. Recent concerns regarding immunogenic responses to systemically administered rAAV at high doses has highlighted the need for investigators to catalog and track potentially immunogenic vector lot components including capsid PTMs and PTMs on host cell protein impurities. Here we present a simple step-by-step guide for academic rAAV laboratories and Chemistry, Manufacturing and Control (CMC) groups in industry to perform an in-house or outsourced bottom-up mass spectrometry workflow to characterize capsid PTMs and process impurities.

## Introduction

Recombinant adeno-associated virus (rAAV) is becoming the most widely used viral vector for gene delivery and genome editing, as it is naturally replication-incompetent, non-lytic, non-pathogenic, and largely non-immunogenic. It exhibits high transduction efficiency in nearly all tissues *in vivo* and can express payloads stably from unintegrated episomes in non-dividing tissues (1). Additionally, it can target integration in actively dividing tissues when homology arms are included in the payload construct (2). Despite decades of use in the clinic, new basic biology of this virus continues to be uncovered. In the last two years alone, we have seen two dogma-changing papers re-shape the AAV textbooks. First, despite the genome’s notoriously small size, yet another new rAAV gene, MAAP, has been found using machine learning (3). MAAP codes for a protein that appears to be involved in rapid extracellular secretion from host cells during production. Second, we reported that during vector production, rAAV genomes are methylated and capsids acquire PTMs (4, 5). These PTMs include acetylation, O-linked glycosylation, phosphorylation, and methylation (in addition to potential degradation products like deamidation and oxidation). Numerous groups have now also independently identified and validated our AAV capsid PTM discoveries (6, 7).

**Figure 1:**
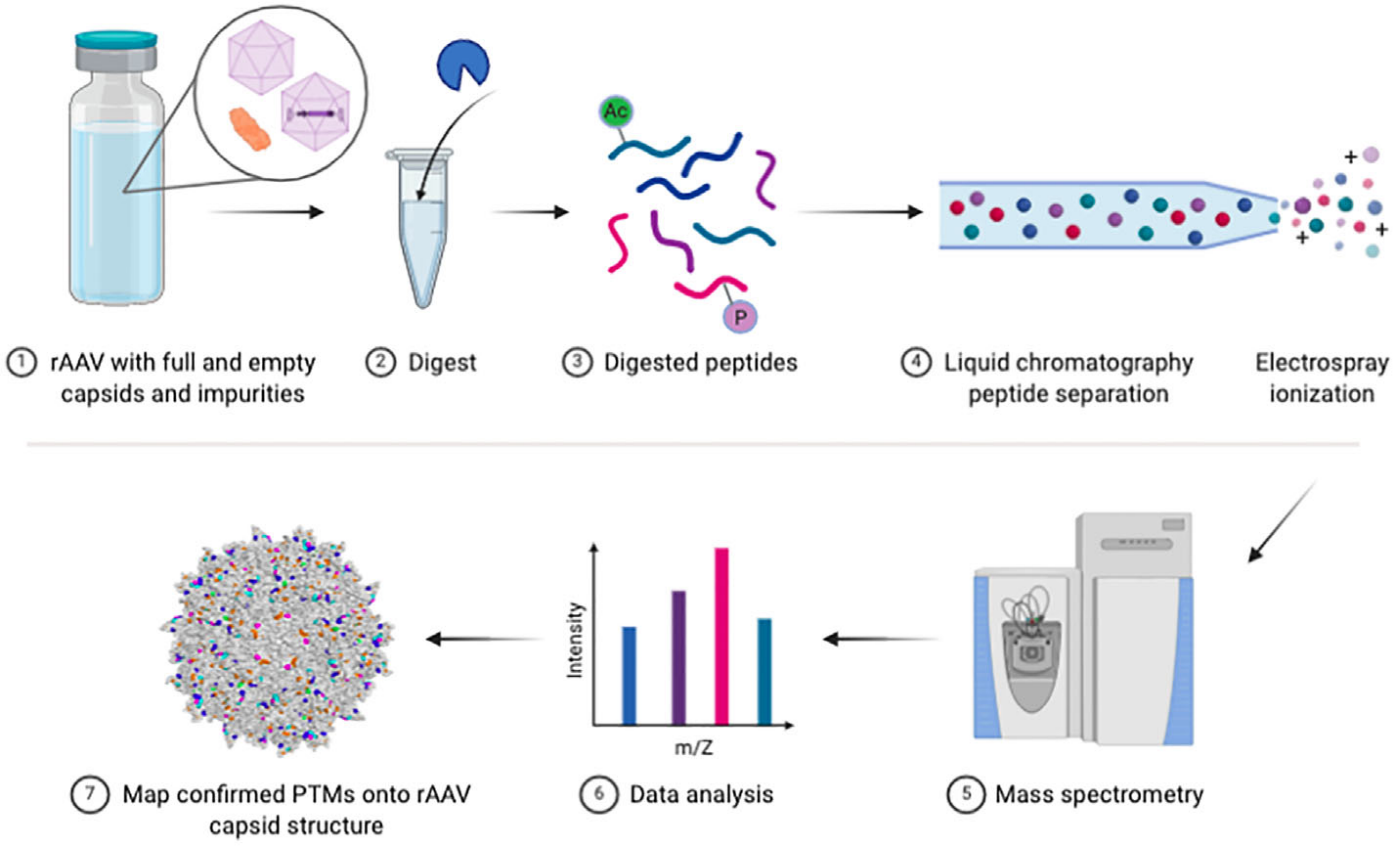
Bottom-up rAAV proteomic workflow. Schematic of key steps in the vector preparation for LC-MS/MS and subsequent data analysis steps.

It is known that PTMs on any therapeutic protein can elicit immune responses by inducing aggregates (8) (a known problem with high concentration rAAV), altering stability or functional activity, or by altering antigen processing and presentation. Despite historically being considered largely non-immunogenic, recent systemically-administered rAAVs at high doses have led to various unwanted immunogenic responses (9–12). It remains unclear what critical quality attributes of rAAV vectors may be contributing to these effects; thus, additional product characterization and preclinical modeling is warranted. Among PTMs, glycosylation specifically can act as a strong modulator of immunogenicity (13). A concern for the rAAV manufacturing space is the potential risk for immunotoxicity from producing vector within insect cells like *Spodoptera frugiperda* in the baculovirus-*Sf9* platform. Humans can have acute allergic responses to non-mammalian N-glycans (14), as well as any N-glycan with an α1,3-fucose or β1,2-xylose linkage on the basal N-acetyl glucosamine (GlcNAc), both of which are modifications found on insect glycoproteins (15). Thus, insect glycoprotein process impurities in baculovirus-*Sf9* produced rAAV vector lots could pose potential immunogenicity risks. As we demonstrated previously (4, 5), rAAV vectors produced in human cells are more potent than vectors produced in insect cells, allowing for lower, potentially safer, doses to be administered. Interestingly, while insect glycoforms can trigger negative human immune responses, conserved mammalian glycoforms (like those that would occur when producing vector in HEK293, HeLa, Vero, or BHK cells) generally enhance product solubility and reduce undesirable immune reactions and aggregation. Glycosylation can also shield potentially immunogenic protein epitopes from the immune system (16). The FDA has existing guidance on what protein PTMs they focus on when reviewing other therapeutic protein products (8, 17, 18), and while these do not yet apply to rAAV, they suggest where the agency may focus as we learn more about the impact these chemical modifications have on rAAV. At present, the FDA lists rAAV capsid PTM assessment as a recommended extended characterization assay (19). Thus, here we provide a detailed protocol for assessing rAAV vector lot PTMs on capsids and host protein impurities (**Figure 1**) based on our years of developing these methods.

## Materials and Equipment

### Reagent List (Vendor, Catalog #)

Protein LoBind 1.5 mL microcentrifuge tubes (Eppendorf Cat#0030108116)
Acetone, liquid chromatography (LC) grade (Thermo Fisher Scientific Cat#AA22928)
Protease Max trypsin enhancer, 1 mg (Promega Cat#V2071)
Ammonium bicarbonate (ABC) (Sigma Cat#09830-500G)
Dithiothreitol (DTT) (Thermo Fisher Scientific Cat#R0861)
Iodoacetamide (Sigma Cat#87-51-4)
Endoglycosidase-H (Endo-H) (Promega Cat#PRV4875)
Sequencing Grade Modified Trypsin (Promega Cat#V5111)
Formic acid, 1 mL ampules (Thermo Fisher Cat#A11710X1-AMP)
C18 MonoSpin SPE columns (GL Sciences Cat#5010-21701)
Acetonitrile (Fisher Scientific Cat#A955-4)
BCA protein assay kit (Pierce Cat#23227)
Bovine serum albumin standard ampules, 2 mg/mL (Pierce Cat#23209)
Millex-GP syringe filter unit 0.22 µm (EMD Millipore Cat#SLGP033RS)
Parafilm (Fisher Scientific Cat#S37441)
Amicon ultra centrifugal filter units, 50 kDa MWCO, 0.5 mL (MilliporeSigma Cat#UFC505008)
Angiotensin I Human Acetate Hydrate (Sigma Aldrich Cat#A9650-1MG)
Vasoactive intestinal peptides fragment VIP 1-12 MS standard (Anaspec Cat#AS-24217)

### Software List

Preview™ and Byonic™ software (Protein Metrics)
XCalibur™ software (Thermo)
Windows-based PC with multi-core processors (8-core AMD) and 8GB of RAM minimum

### Equipment List

Refrigerated benchtop centrifuge (any manufacturer)
Centrifugal vacuum concentrator (SpeedVac or any manufacturer)
Microcentrifuge (any manufacturer)
ACQUITY UPLC M-Class System (Waters Corporation)
Orbitrap Fusion™ Tribrid™ Mass Spectrometer (Thermo Fisher Scientific)
Dionex Ultimate 3000 HPLC (Thermo Fisher Scientific)
75 µm x 150 mm EASY-Spray™ column 2 µM C18 beads (Thermo Scientific Cat#ES904)
Autosampler (Thermo Fisher Scientific)
Vortexer (any manufacturer)
Thermomixer (Eppendorf)

### Solution Formulations

#### Solutions to Prepare in Advance and Store

100 mM ammonium bicarbonate (ABC)

Combine all reagents listed and bring to a final volume of 200 mL. Sterile filter with a 0.22 µm filter and store at room temp.

**Table:** 

Reagent	[Final]	Amount needed
NH_4_HCO_3_ (MW: 79.06 g/mol)	100 mM	1.58 g
Distilled water	–	As needed (~198 mL)
Total Volume	–	200 mL

#### Solutions to Prepare Fresh

100-mM dithiothreitol (DTT)

Combine all reagents listed and bring to a final volume of 100 mL. Sterile filter with a 0.22 µm filter and store at 4°C.

**Table:** 

Reagent	[Final]	Amount needed
C_4_H_10_O_2_S_2_ (MW: 154.25 g/mol)	100 mM	1.54 g
Distilled water	–	As needed (~98 mL)
Total Volume	–	100 mL

*CAUTION: Dithiothreitol (DTT) is a harmful chemical and should be handled with appropriate safety precautions in a chemical fume hood. When handling DTT stock, wear appropriate PPE including a lab coat, goggles/face shield, closed toe shoes and gloves. Dispose of materials that contact DTT in a labeled solid waste container (weigh boats, pipette tips, etc.).

## Methods

### Proteolytic Digests of rAAV Samples for LC-MS/MS

#### Time Required: 2.5 Days

1. Determine the protein concentration of your rAAV samples. We strongly recommend using the colorimetric bicinchoninic acid (BCA) assay as it is fast, accurate, and has the largest relevant dynamic range (20-2,000 µg/ml) suitable for AAV concentrations compared to other common assays like Bradford, Lowry, and NanoOrange. We prefer the Pierce BCA kit and recommend following the manufacturer’s instructions for the ‘Standard’ assay rather than the ‘Enhanced’ assay for a more relevant working range of the bovine serum albumin controls. Read all assay results simultaneously on a microplate reader, rather than one-by-one on a spectrophotometer, given the rapid colorimetric change and large number of controls, samples, and replicates.
*Stop point: you can keep your thawed AAV at 4°C until you are ready to continue. Although AAV is stable at 4°C short term, we recommend preparing your samples for digestion as soon as possible after thawing and performing the BCA assay.
2. The desired amount of total protein per rAAV sample is 50 μg, but we’ve successfully run samples with as little as 10 μg. Whatever concentration you choose, if you are running multiple rAAV samples with the intention of comparing the results, prepare an equal amount of total protein for each sample and place each in a labeled protein low-binding 1.5 mL microcentrifuge tube.
3. Precipitate each rAAV sample in 4X the volume of LC-grade acetone overnight at -80°C with the tube wrapped in parafilm.
4. To separate out the precipitated rAAV proteins, centrifuge at 12,500 *x g* for 15 min at 4°C. Decant and discard the supernatant and dry the rAAV protein pellet within a chemical safety hood for 30 min with the tube lid open.
5. Resuspend the dried pellet in 100 μL of 0.2% Protease Max surfactant trypsin enhancer*, 50 mM ammonium bicarbonate, and reduce the disulfide bonds with dithiothreitol to a final concentration of 10 mM at 55°C for 30 min in a thermomixer. This step loosens the protein secondary structure to make the full-length polypeptide accessible for enzymolysis in the next step.
* Note: Protease Max surfactant helps solubilize proteins. This will help your AAV stay in solution, but it is not strictly necessary for digestion, should it be difficult to acquire.

**Table:** 

Reagent	[Stock]	[Final]	Volume needed (µL)
Dried total protein pellet with rAAV	–	10-50 μg	Dried pellet
Protease Max trypsin enhancer	1%	0.2%	20
Ammonium bicarbonate (ABC)	100 mM	50 mM	50
Dithiothreitol (DTT)	100 mM	10 mM	10
Distilled water			As needed (~20)
Total volume	–	–	100
*CAUTION: Dithiothreitol (DTT) is a harmful chemical and should be handled with appropriate safety precautions in a chemical fume hood. When handling DTT stock, wear appropriate PPE including a lab coat, goggles/face shield, closed toe shoes and gloves. Dispose of materials that contact DTT in a labeled solid waste container (weigh boats, pipette tips, etc.).
6. Following reduction of the disulfide bonds, alkylate the now-free sulfhydryl groups with an alkylating agent (e.g. 20 mM propionamide or iodoacetamide) at 25°C for 30 min in the dark. This step permanently prevents the reformation of disulfide bonds, keeping the polypeptide accessible.
7. To digest full-length rAAV polypeptides into short peptides, add 500 ng of Trypsin, wrap the tubes in parafilm to prevent evaporation, and allow the solution to digest overnight for ~18 h at 37°C in a thermomixer. Other digestion enzymes can be used in place of or in combination with Trypsin for varied digestion specificity (e.g. Chymotrypsin, etc.).
8. To stop the protease activity, add 1% total volume formic acid to the reaction in a chemical fume hood.
*CAUTION: Formic acid is a harmful chemical and should be handled with appropriate safety precautions in a chemical fume hood. When handling formic acid at any concentration, wear appropriate PPE including a lab coat, goggles/face shield, closed toe shoes and gloves. Dispose of materials that contact formic acid in a labeled solid waste container (weigh boats, pipette tips, etc.). Store formic acid at 4°C.
*Stop point: you can keep your quenched AAV peptides at -20°C for long term storage or 4°C for clean up the next day.
9. Purify the digested peptides by running the solution through a C18 MonoSpin SPE column, and then dry the peptides to completion in a speed vac. The speed vac time needed will vary with the elution volume. For example, a 300 μL solution with 80% acetonitrile can take 4-6 h.

### Liquid Chromatography Tandem Mass Spectrometry

#### Time Required: 1.5 h

1. Reconstitute your dried rAAV peptides in 0.1% formic acid in ultrapure water and then inject onto an HPLC system such as a Dionex Ultimate 3000.
*CAUTION: Formic acid is a harmful chemical and should be handled with appropriate safety precautions in a chemical fume hood. When handling formic acid at any concentration, wear appropriate PPE including a lab coat, goggles/face shield, closed toe shoes and gloves. Dispose of materials that contact formic acid in a labeled solid waste container (eppendorf tubes, pipette tips, etc.).
*CAUTION: Acetonitrile is a highly dangerous chemical that is flammable in both liquid and vapor phases and can ignite with moist air or water. Is harmful if swallowed, inhaled, or absorbed through the skin. May cause skin and respiratory tract irritation. It is metabolized to cyanide in the body, which may cause headache, dizziness, weakness, unconsciousness, convulsions, coma and possibly death. Use only in explosion-proof chemical fume hoods equipped with proper grounding procedures to avoid static electricity.
2. We recommend running rAAV samples on a Thermo Fisher Fusion™ Tribrid™ Series or Q-Exactive™ mass spectrometer to improve proteome coverage, detection limits, peptide spectra acquisition, and identification rates. Unlike a linear hybrid model, which combines an ion trap and an Orbitrap, a parallelized tribrid combines a quadrupole, Orbitrap, and linear ion trap. We prefer the Orbitrap Fusion™ Tribrid™ or the Orbitrap Fusion™ Lumos™ Tribrid™ to acquire PTM data. Set up the instrument to acquire data in a dependent fashion using higher-energy collisional dissociation (HCD). If you are going to be analyzing labile modifications such as phosphorylation or glycosylation, we recommend also using electron-transfer dissociation (ETD) (20). The instrument parameters we suggest are as described below.
3. Generate HCD data with the precursor mass resolution set to 60,000 at full width at half maximum 400 *m/z*, a mass range of 350-1,500 *m*/*z*, and sample charge states 2-6. Set the precursor automated gain control (AGC) settings to 3e5 ions, and use the “fastest” mode in the MS/MS ion trap. Set the isolation window for HCD to 1.6 Da and the collision energy to 30. Enable dynamic exclusion with a repeat count of 3, repeat duration of 10 sec, and an exclusion duration of 10 sec. MS2 spectra should be generated at top speed for 3 sec.
4. If you are analyzing labile PTMs such as glycosylation, set up a product-dependent HCD-triggered-ETD method. Here, perform ETD if: (a) the precursor mass is between 300 and 1000 *m/z* and (b) in the HCD spectrum, 3 of 7 glyco-fingerprint ions (126.055, 138.055, 144.07, 168.065, 186.076, 204.086, 274.092, 292.103) are present at +/- 0.1 *m/z* and greater than 5% relative intensity. Set ETD parameters as follows: calibrated charge-dependent ETD times, 2e5 reagent target, and precursor AGC target 1e4. Read out fragment ions in the ion trap in a centroid fashion.
5. Prior to running your rAAV samples, we recommend running calibration standards to ensure the instrument is within mass tolerance. We prefer to use Angiotensin I and Anaspec VIP (1–12) MS standards. Reconstitute to 500 fmol/µL and inject 1 µl to assure that calibration is within 5 ppm prior to injecting your rAAV samples.
  Angiotensin I standard: monoisotopic molecular weight of 1296.6848 Da
  VIP (1-12) standard: monoisotopic molecular weight of 1425.6393 Da
6. Load samples *via* autosampler and inject using a flow rate of 0.3 µL/min onto a 75 µm x 150 mm EASY-Spray column containing 2 µm C18 beads. Hold columns at 40°C using a column heater in the EASY-Spray ionization source.
**Note:** This setup can be variable and tailored to fit your rAAV sample. The exact HPLC and column type can be adjusted and optimized for your sample separation.
7. Chromatographically separate the sample using a 90 min gradient and a 140 min instrument method. Solvent A should be 0.1% formic acid in ultrapure water, and Solvent B should be 0.1% formic acid in acetonitrile. Setup the gradient profile as follows: (minute:%B):

**Table:** 

Minute	% Solvent B
0	3
3	3
93	35
103	42
104	98
109	98
110	3
140	3
8. Following your final rAAV sample, re-run your calibration standards as before (see Step #1) to ensure the instrument is still within tolerance at the end of your rAAV sample run.

## Anticipated Results

### Mass Spectra Data Analysis Using Byonic

**Figure 2:**
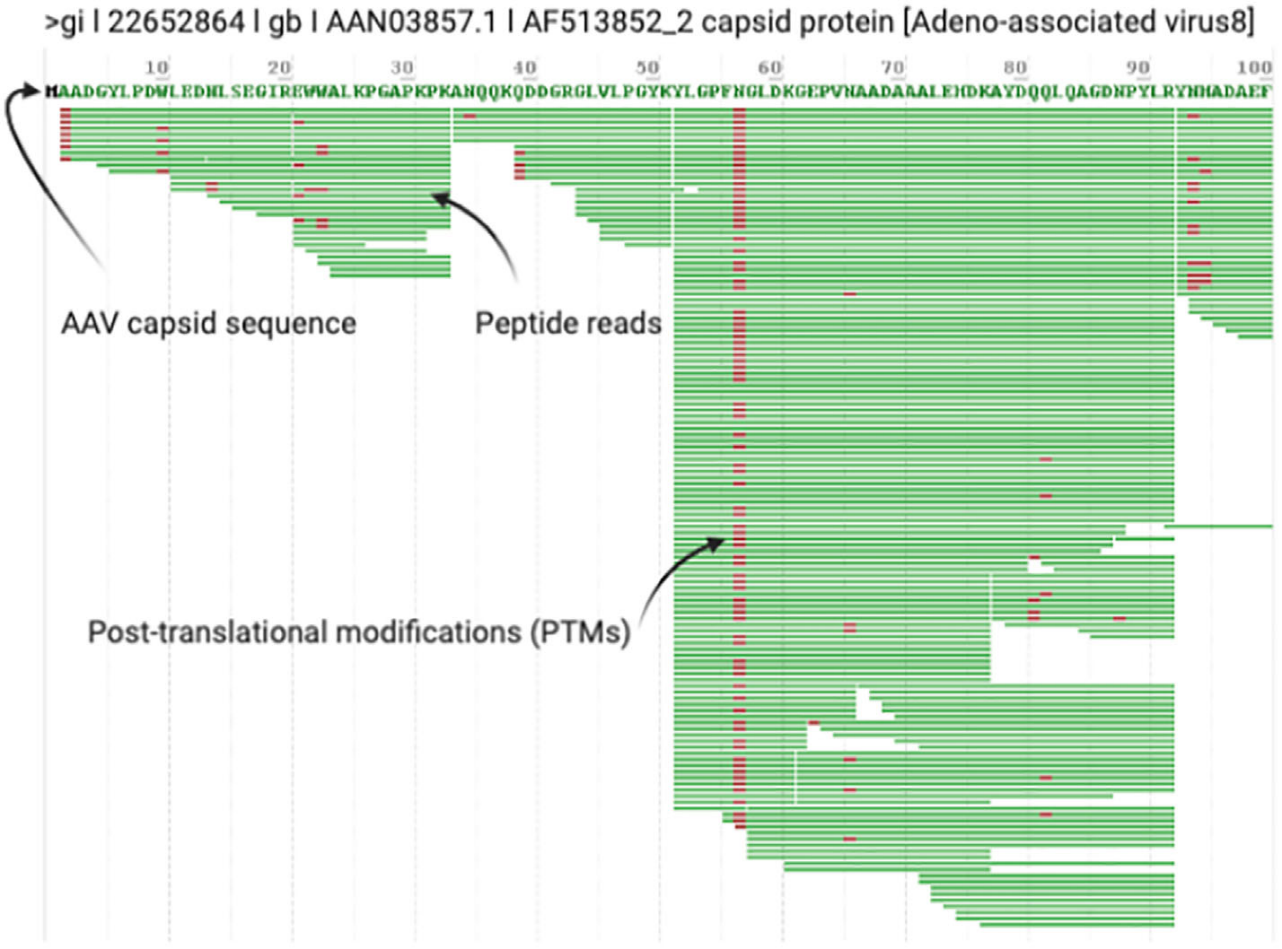
Example Digestion Efficiency Validation. Trypsin cleaves peptides on the C-terminal side of lysine and arginine residues (unless followed by a proline). All rAAV capsid serotypes have regular Lys and Arg amino acids throughout VP1 so you should see regular cleavage that produces peptides ~10-45 amino acids in length (shown in Preview as green bars with PTMs noted in red) throughout the entire length of VP1. Good digestion efficiency and MS/MS runs should exhibit >90% sequence coverage of rAAV VP1. This screenshot highlights typical results for the first 100 amino acids of a prototypical rAAV8 run.

1. Prepare a concatenated FASTA file containing the relevant protein sequences for your rAAV capsid serotype and host proteome(s): *Homo sapiens* for HEK293 and HeLa cells, *Spodoptera frugiperda* for *Sf9* cells, *Chlorocebus aethiops* for Vero cells, *Mesocricetus auratus* for BHK cells, *Autographa californica multiple nuclepolyhedrovirus* for baculovirus, and *Adenoviridae* or *Herpesviridae* if using Adenovirus or HSV-1 to rescue stable lines*, etc.* You can also prepare an exclusion list for common contaminants (21, 22) that typically originate from the user (e.g. keratin from hair and skin), and from common reagents (e.g. Trypsin) used in sample preparation.
2. Search your raw files with 10–12 ppm mass tolerances for precursor mass ions, with 10–12 ppm or 0.1–0.4 Da fragment mass tolerances for HCD and ETD fragmentation, respectively. Allow up to two missed cleavages per peptide and semi-specific, N-ragged tryptic digestion. Use a 1% false discovery rate using standard reverse-decoy techniques. Methionine oxidation (common 2), asparagine deamidation (common 2), and N-term acetylation (rare 1) should be set as variable modifications with a total common max of 3, rare max of 1. Depending on the goals of your experiment, you may also want to add phosphorylation, methylation, acetylation, and/or glycosylation as variable modifications. Note that the search time will increase exponentially with additional modifications, so it may be advantageous to search these modifications separately.
3. After the search is complete, open the.RAW file in the ProteinMetrics Preview software to view the trypsin digestion efficiency (**Figure 2**). The resulting identified peptide spectral matches and assigned proteins should then be exported for further analysis and validated using custom tools to provide visualization and statistical characterization.
4. PTM mass spectra should be manually validated by an expert. Do not simply trust PTM reports produced by the software. If you are not skilled in MS analysis of PTMs, seek help from an expert. Here we refer readers to an excellent manuscript summarizing common errors (23) we’ve seen among groups incorrectly interpreting PTMs in rAAV vector lots:
  - Assigning the wrong PTM to a peptide
  - Assigning the correct PTM to the wrong protein
  - Assigning a PTM to the wrong residue on a correctly identified peptide
  - Missing modified peptides because of a flawed database search strategy

### *De Novo* Glycan Identification

**Figure 3:**
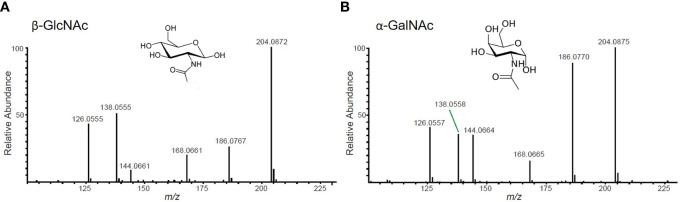
Representative HexNAc fingerprints. **(A)** GlcNAc-containing glycopeptides will have a higher abundance of the ion at 138 *m/z* than the ion at 144 *m/z*. This fingerprint will be present in peptides with N-glycans, O-GlcNAc, and/or GlcNAc-containing core 2 O-glycans. **(B)** GalNAc-containing glycopeptides will have nearly equal abundance of 138 *m/z* and 144 *m/z* ions. This fingerprint will be present in mucin-type glycopeptides that do not contain GlcNAc.

1. All potential glycopeptide sequences should be validated by *de novo* manual interpretation of HCD and ETD mass spectra. For a thorough guide on this subject, please see Malaker et al. (24). Briefly, generate extracted chromatograms for all MS2 spectra containing the “HexNAc fingerprint,” which consists of a 204.0867 *m/z* ion and 5 additional fragment ions.
2. First, use the HCD spectrum containing the HexNAc fingerprint to identify glycan structures. Distinguish whether the glycopeptide is modified by an N- or O-glycan by analyzing the ratio of 138 *m/z* to 144 *m/z* ions (**Figure 3**). Then, calculate the intact mass of the glycopeptide using the high-resolution MS1 spectrum. From the intact mass, you will see sequential glycan losses that will lead to the largest peak in the spectrum. For N-glycopeptides, this will be the mass of the peptide modified by one HexNAc. For O-glycopeptides, this will be the mass of the naked peptide backbone. From this, you can calculate the glycan monosaccharide composition. Finally, sequence the peptide backbone using techniques described in detail elsewhere (25).
3. Next, use the ETD spectrum to site-localize glycan modifications. This is especially important for O-glycopeptides, due to the labile nature of this modification. A detailed tutorial for manual interpretation of ETD spectra is available here (26).

## Troubleshooting

1. **Your purified input rAAV is not sufficiently concentrated to achieve 10-50 μg in 50 μL volume.**
  If your purified rAAV production has a low titer, below ~1e12 vg/mL, you will likely not have sufficient rAAV capsid protein concentration for the initial digestion and subsequent steps. You can concentrate your rAAV vector with an Amicon™ 50 kDa spin column. We recommend a 20-30 minute spin at 1,500 *x g* for ~10-20X concentration as suggested by the manufacturer for purified AAV (27). You will need to remeasure your protein concentration by BCA assay after completing the spin concentration, do not assume you will get a certain fold improvement. Make sure to use the 50 kDa Amicon™ columns for optimal rAAV retention as intact rAAV capsids are 20-24 nm in diameter (28), as shown below.

**Table:** 

MWCO (kDa)	Pore Size (nm)	Min Retention Diameter (nm)	Max Retention Diameter (nm)
3	0.3	1	1.5
10	1	3	9
30	3	9	15
**50**	**5**	**15**	**30**
100	10	30	90
2. **Difficulty re-solubilizing your rAAV proteins.**
  As noted above, surfactant may not be necessary for protein resolubilization; however, if rAAV protein samples are insoluble in ABC buffer, two distinct steps can be followed to improve sample solubility. First, add a mass spectrometry friendly surfactant, such as Protease Max, to the samples and gently mix by pipette action. Incubate the samples at room temperature for 20 min before re-mixing. If this does not solve the problem, samples can be heated using a heating block set to 95°C for up to 5 min.
3. **AAV sample degradation from freeze/thaw.**
  For long term native AAV storage, samples should be kept at -80°C, however digested rAAV peptides can be stored at -80°C. This will prevent rAAV VP1/2/3 peptide degradation and help to retain labile PTMs. For short-term storage once thawed, samples can be kept at 4°C; however, it is important to avoid repeated freeze/thaw cycles, as the extreme temperature fluctuation can lead to more rapid sample degradation. We typically avoid more than 1-2 freeze/thaw cycles per sample prior to analysis.
4. **Contaminating peptides obscuring your results.**
  Perhaps one of the biggest complicating factors in analyzing rAAV samples is the potential presence of contaminating peptides in your sample. Notably, these peptides might also have PTMs that can interfere with the analysis of your rAAV sample. Contaminant peptides can come from a variety of sources, including sample handling, host cell impurities, and vector purification inefficiencies. Therefore, it is crucial to manually validate all search results to ensure the correct site-localization of PTMs on the proper protein. We recommend searching for not only the sequence of interest (i.e. rAAV of the appropriate serotype), but also the proteomes of potentially contaminating species, such as *Sf9* or other sources involved in vector production. In our hands, upon manual validation we found several hundred N-glycopeptides from ferritin proteins that were initially assigned as N-glycopeptides of rAAV. This underscores the importance of carefully manually validating automated search results by the methods outlined above.
5. **If you think your sample has glycopeptides but you are having trouble detecting them.**
  Following the reduction of disulfide bonds in Step 6, treat with Endo-H. Heat your reduced rAAV sample to 95°C for 5 min, then briefly chill on ice to reduce the temperature and add 5 μL of the supplied Endo-H reaction buffer and 5 μL Endo-H. Deglycosylate for 4 h at 37°C in a thermomixer.
*Note: Endo-H is a recombinant glycosidase which hydrolyses the bond connecting the two GlcNAc groups modifying Asn within the chitobiose core, leaving a single GlcNAc covalently bound to Asn for mass spectrometry detection.
6. **Sample analysis challenges and limitations.**
  With all PTM assignments using LC-MS/MS, it is important to remember that absence of a peptide with a PTM does not mean that no PTM was present. Labile modifications like phosphorylation and glycosylation can be lost during sample preparation or ionization prior to detection. It is also important to note that quantifying PTM frequency is challenging. In many cases, PTMs are not site-localizable, which can dramatically alter the accuracy of quantitative site occupancy evaluations. Additionally, different peptides from an individual protein can differ in cleavage efficiency.

## Discussion

Preclinical investigators and existing clinical stage gene therapy companies should catalog and track potentially immunogenic vector lot components. These include capsid PTMs and PTMs on host cell protein impurities, especially given the recent concerns around immunogenicity with systemic high dose rAAV. The FDA currently lists rAAV capsid PTM assessment as a recommended extended characterization assay (19). The methods detailed above provide a powerful platform to easily interrogate the proteomic and PTM landscapes of rAAV vectors. As improvements to both vector process/product development and analysis by mass spectrometry continue, the quality, complexity, and utility of these data will continue to grow. These methods can be extended to other viral gene therapy vectors by changing the appropriate search parameters and optimizing the initial proteolytic digest conditions (for example, enveloped viruses will need a detergent step to remove the envelope to allow proteases to digest the capsid proteins). While we regularly detect common PTMs on rAAV capsids and host impurities (N-terminal start methionine acetylation, serine/threonine/tyrosine phosphorylation, lysine acetylation, arginine methylation, O-linked glycosylation, and asparagine deamidation), other modifications, if present, can also be detected with this method.

## Data Availability Statement

The data analyzed in this study is subject to the following licenses/restrictions: exemplary datasets analyzed for this method are available from the corresponding authors on reasonable request. Requests to access these datasets should be directed to NKP, nicole.paulk@ucsf.edu.

## Author Contributions

NKP conceived the study. NGR, SAM, and NKP designed experiments. NGR, SAM, and NKP generated reagents, protocols, performed experiments, and analyzed data. NGR, SAM, and NKP wrote the manuscript. SAM and NKP generated the figures. All authors contributed to the article and approved the submitted version.

## Funding

This research was supported by grants to NKP from the NIH (K01-DK107607, U01-HL145795), an American Society of Gene & Cell Therapy Career Development Award and the Sandler Family Foundation; and funds to SAM from the Yale Science Development Fund. The contents of this publication are solely the responsibility of the authors and do not necessarily represent the official views of the funding bodies, or the respective universities and organizations.

## Conflict of Interest

The authors declare that the research was conducted in the absence of any commercial or financial relationships that could be construed as a potential conflict of interest.

## References

[1] Nakai H Yant SR Storm TA Fuess S Meuse L Kay MA. Extrachromosomal recombinant adeno-associated virus vector genomes are primarily responsible for stable liver transduction in vivo. J Virol (2001) 75(15):6969–76. 10.1128/JVI.75.15.6969-6976.2001 PMC114425 11435577

[2] Alexander IE Russell DW Spence AM Miller AD. Effects of gamma irradiation on the transduction of dividing and nondividing cells in brain and muscle of rats by adeno-associated virus vectors. Hum Gene Ther (1996) 7(7):841–50. 10.1089/hum.1996.7.7-841 8860836

[3] Ogden PJ Kelsic ED Sinai S Church GM. Comprehensive AAV capsid fitness landscape reveals a viral gene and enables machine-guided design. Science (2019) 366:1139–43. 10.1126/science.aaw2900 PMC7197022 31780559

[4] Rumachik NG Malaker SA Poweleit N Maynard LH Adams CM Leib RD. Methods Matter – Standard Production Platforms For Recombinant AAV Can Produce Chemically And Functionally Distinct Vectors. BioRxiv (2019) 640169. 10.1101/640169v1.abstract PMC7488757 32995354

[5] Rumachik NG Malaker SA Poweleit N Maynard LH Adams CM Leib RD. Methods Matter: Standard Production Platforms for Recombinant AAV Produce Chemically and Functionally Distinct Vectors. Mol Ther - Methods Clin Dev (2020) 18:98–118. 10.1016/j.omtm.2020.05.018 32995354 PMC7488757

[6] Liu AP Patel SK Xing T Yan Y Wang S Li N. Characterization of Adeno-Associated Virus Capsid Proteins Using Hydrophilic Interaction Chromatography Coupled with Mass Spectrometry. J Pharm Biomed Anal (2020) 189:113481. 10.1016/j.jpba.2020.113481 32750536

[7] Mary B Maurya S Arumugam S Kumar V Jayandharan GR. Post-translational modifications in capsid proteins of recombinant adeno-associated virus ( AAV ) 1-rh10 serotypes. FEBS J (2019) 286:4964–81. 10.1111/febs.15013 PMC7496479 31330090

[8] Center for Drug Evaluation, Research. Immunogenicity Assessment for Therapeutic Protein Products. (2020). Available at: https://www.fda.gov/regulatory-information/search-fda-guidance-documents/immunogenicity-assessment-therapeutic-protein-products.

[9] Paulk NK. Gene Therapy: It"s Time to Talk about High-Dose AAV (2020). Available at: https://www.genengnews.com/commentary/gene-therapy-its-time-to-talk-about-high-dose-aav.

[10] Wilson JM Flotte TR. Moving forward after two deaths in a gene therapy trial of myotubular myopathy. Hum Gene Ther (2020) 31(13-14):695–6. 10.1089/hum.2020.182 32605399

[11] Srivastava A Vectors AAV. Are They Safe? Hum Gene Ther (2020) 31(13-14):697–9. 10.1089/hum.2020.187 32611206

[12] Shieh PB Bönnemann CG Müller-Felber W Blaschek A Dowling JJ Kuntz NL. Re: “Moving Forward After Two Deaths in a Gene Therapy Trial of Myotubular Myopathy” by Wilson and Flotte. Hum Gene Ther (2020) 31(15-16):787. 10.1089/hum.2020.217 32777938 PMC7462017

[13] Malaker SA Penny SA Steadman LG Myers PT Loke JC Raghavan M. Identification of Glycopeptides as Posttranslationally Modified Neoantigens in Leukemia. Cancer Immunol Res (2017) 5:376–84. 10.1158/2326-6066.cir-16-0280 PMC5508727 28314751

[14] Altmann F. The Role of Protein Glycosylation in Allergy. IAA (2007) 142(2):99–115. 10.1159/000096114 17033195

[15] Minagawa S Sekiguchi S Nakaso Y Tomita M Takahisa M Yasuda H. Identification of Core Alpha 1,3-Fucosyltransferase Gene From Silkworm: An Insect Popularly Used to Express Mammalian Proteins. J Insect Sci (2015) 15:110. 10.1093/jisesa/iev088 26223947 PMC4675719

[16] Wei X Decker JM Wang S Hui H Kappes JC Wu X. Antibody neutralization and escape by HIV-1. Nature (2003) 422(6929):307–12. 10.1038/nature01470 12646921

[17] Center for Drug Evaluation, Research. Development of Therapeutic Protein Biosimilars: Comparative Analytica. (2020). Available at: https://www.fda.gov/regulatory-information/search-fda-guidance-documents/development-therapeutic-protein-biosimilars-comparative-analytical-assessment-and-other-quality.

[18] Center for Drug Evaluation, Research. Quality Considerations in Demonstrating Biosimilarity of a Therapeutic. (2020). Available at: https://www.fda.gov/regulatory-information/search-fda-guidance-documents/quality-considerations-demonstrating-biosimilarity-therapeutic-protein-product-reference-product.

[19] Byrne A. Chief, Gene Transfer and Immunogenicity Branch, Division of Cellular and Gene Therapies, FDA. Standardization in Vector Manufacturing. In: Session: Challenges and Solutions in Large-Scale Manufacturing. (2020). ASGCT Policy Summit. Available at: https://www.asgct.org/advocacy/policy-summit/2020-agenda.

[20] Riley NM Malaker SA Driessen MD Bertozzi CR. Optimal Dissociation Methods Differ for - and -Glycopeptides. J Proteome Res (2020) 19(8):3286–301. 10.1021/acs.jproteome.0c00218 PMC7425838 32500713

[21] Mellacheruvu D Wright Z Couzens AL Lambert J-P St-Denis N Li T. The CRAPome: a Contaminant Repository for Affinity Purification Mass Spectrometry Data. Nat Methods (2013) 10(8):730. 10.3410/f.718060933.793484725 23921808 PMC3773500

[22] Hodge K Ten Have S Hutton L Lamond AI. Cleaning up the masses: Exclusion lists to reduce contamination with HPLC-MS/MS. J Proteomics (2013) 88:92. 10.1016/j.jprot.2013.02.023 23501838 PMC3714598

[23] Kim M-S Zhong J Pandey A. Common errors in mass spectrometry-based analysis of post-translational modifications. Proteomics (2016) 16(5):700–14. 10.1002/pmic.201500355 PMC5548100 26667783

[24] Malaker SA Ferracane MJ. Mass Spectrometric Identification and Molecular Modeling of Glycopeptides Presented by MHC Class I and II Processing Pathways. In: Immunoproteomics. New York, NY: Humana (2019). p. 269–85. 10.1007/978-1-4939-9597-4_17 31364056

[25] Papayannopoulos IA. The interpretation of collision‐induced dissociation tandem mass spectra of peptides. Mass Spectrom Rev (1995) 14:49–73. 10.1002/mas.1280140104

[26] Electron Transfer Dissociation (2021). Available at: http://people.virginia.edu/~dlb6z/HuntETDTutorial/ETD_Page1test.html1.

[27] Virus Concentration by Ultrafiltration (2020). Available at: https://www.sigmaaldrich.com/technical-documents/protocols/biology/viral-concentration-amicon-ultrafiltration.html.

[28] Steinbach S Wistuba A Bock T Kleinschmidt JA. Assembly of adeno-associated virus type 2 capsids in vitro. J Gen Virol (1997) 78( Pt 6):1453–62. 10.1099/0022-1317-78-6-1453 9191943

